# Treatment of Benign Prostatic Hyperplasia by Natural Drugs

**DOI:** 10.3390/molecules26237141

**Published:** 2021-11-25

**Authors:** Eszter Csikós, Adrienn Horváth, Kamilla Ács, Nóra Papp, Viktória Lilla Balázs, Marija Sollner Dolenc, Maša Kenda, Nina Kočevar Glavač, Milan Nagy, Michele Protti, Laura Mercolini, Györgyi Horváth, Ágnes Farkas, on behalf of the OEMONOM

**Affiliations:** 1 Department of Pharmacognosy, Faculty of Pharmacy, University of Pécs, H-7624 Pécs, Hungary; csikos.eszter@gytk.pte.hu (E.C.); kamilla.acs@gmail.com (K.Á.); nora4595@gamma.ttk.pte.hu (N.P.); viktoria.balazs@aok.pte.hu (V.L.B.); horvath.gyorgyi@gytk.pte.hu (G.H.); 2 Department of Pharmaceutical Biology, Faculty of Pharmacy, University of Pécs, H-7624 Pécs, Hungary; horvath.adrienn2@pte.hu; 3 University of Ljubljana, Department of Pharmaceutical Chemistry, Faculty of Pharmacy, SI-1000 Ljubljana, Slovenia; marija.sollner@ffa.uni-lj.si (M.S.D.); masa.kenda@ffa.uni-lj.si (M.K.); 4 University of Ljubljana, Department of Pharmaceutical Biology, Faculty of Pharmacy, SI-1000 Ljubljana, Slovenia; nina.kocevar.glavac@ffa.uni-lj.si; 5 Department of Pharmacognosy and Botany, Faculty of Pharmacy, Comenius University in Bratislava, SK-832-32 Bratislava, Slovakia; milan.nagy@fpharm.uniba.sk; 6 Research Group of Pharmaco-Toxicological Analysis (PTA Lab), Department of Pharmacy and Biotechnology (FaBiT), Alma Mater Studiorum—University of Bologna, 40126 Bologna, Italy; michele.protti2@unibo.it (M.P.); laura.mercolini@unibo.it (L.M.)

**Keywords:** benign prostatic hyperplasia, medicinal plants, phytotherapy, saw palmetto, preclinical studies, clinical efficacy, safety issues

## Abstract

Benign prostatic hyperplasia (BPH) is one of the most common urinary diseases affecting men, generally after the age of 50. The prevalence of this multifactorial disease increases with age. With aging, the plasma level of testosterone decreases, as well as the testosterone/estrogen ratio, resulting in increased estrogen activity, which may facilitate the hyperplasia of the prostate cells. Another theory focuses on dihydrotestosterone (DHT) and the activity of the enzyme 5α-reductase, which converts testosterone to DHT. In older men, the activity of this enzyme increases, leading to a decreased testosterone/DHT ratio. DHT may promote prostate cell growth, resulting in hyperplasia. Some medicinal plants and their compounds act by modulating this enzyme, and have the above-mentioned targets. This review focuses on herbal drugs that are most widely used in the treatment of BPH, including pumpkin seed, willow herb, tomato, maritime pine bark, Pygeum africanum bark, rye pollen, saw palmetto fruit, and nettle root, highlighting the latest results of preclinical and clinical studies, as well as safety issues. In addition, the pharmaceutical care and other therapeutic options of BPH, including pharmacotherapy and surgical options, are discussed, summarizing and comparing the advantages and disadvantages of each therapy.

## 1. Introduction

Benign prostatic hyperplasia (BPH) is one of the most common urinary diseases in aging men, which can lead to lower urinary tract symptoms (LUTS). One of the most important risk factors in the occurrence of the pathology of BPH is age. The prevalence of the disease increases with age. Other specific risk factors are prostate volume (PV), LUTS, and serum prostate-specific antigen (PSA) [1]. A total of 30% of men over the age of 65 could be affected by LUTS [2]. The most predictive symptoms are nocturia and changes in urinary flow stream. An enlarged prostate and LUTS together form complex symptoms. Firstly, there is a static component, which is the direct bladder outlet obstruction (BOO) from the enlarged tissue. Secondly, there is a dynamic component from the increased smooth muscle tone of the bladder neck [3]. The obstructive symptoms include, for example, urinary flow intermittency, and a sense of incomplete bladder emptying due to urinary retention, or terminal dribbling. The static component causes irritative symptoms, such as incontinence, nocturia, or hematuria [4]. Moderate and severe urinary symptoms can greatly impair a patient’s quality of life. The origin of BPH is not exactly known, but three theories have been proposed: (1) the prostate cells can convert approximately 90% of testosterone to dihydrotestosterone (DHT) by 5α-reductase. The DHT has a higher affinity to androgen receptors, and seems to stimulate protein synthesis, differentiation, and prostate cell growth [5,6]; (2) The second theory about BPH development is based on the prostate cells, which are androgen-independent and can self-renew in androgen-deficient conditions [7]; (3) The third is based on the interactions between stroma and epithelium. Both of them can convert testosterone into DHT. This process allows the production of various growth factors [7,8].

Depending on the severity of the disease, there are different types of treatment options. Herbal remedies, medicines, and surgery are available. BPH in mild to moderate cases can be treated with herbal remedies. In more severe cases, medicines may be given. Several herbal preparations in this indication are easily available to patients. The most common active ingredients they contain are phytosterols, β-sitosterol, lectins, etc. [9].

## 2. Medical Therapy of BPH

European and non-European guidelines focusing on therapeutic options of the disease include pharmacotherapy, lifestyle recommendations, surgical options, and phytotherapy, as well [10,11,12,13]. In this chapter, we primarily discuss prescription drug therapies and surgical possibilities in more detail.

The diagnosis of BPH involves specialist skills, based on the results of the patient’s symptoms, the laboratory, and other tests. Completing the International Prostate Symptom Score (IPSS) could be useful in assessing the severity of the disease and choosing the right therapy. Based on the results of the questionnaire, patients can be classified into mild (IPSS: 0–7), moderate, and severe (IPSS: 20–35) categories [10].

All patients with LUTS should be offered lifestyle advice before (or in combination with) medical treatment. Those whose lifestyles are not impaired by their symptoms can manage with conservative treatment, which is called watchful waiting (WW). WW includes patient education, monitoring, and lifestyle recommendations. In this case, patients should focus on the reduction of fluid intake (especially during the night and while traveling), and avoiding diuretic and irritant agents (e.g., caffeine, alcohol) [14]. The use of relaxation and urination techniques (e.g., double-voiding, perineal pressure, the urethral milking technique, breathing exercises, mental tricks, etc.) help control storage symptoms [10,15]. In the mild or moderate categories, WW can be supplemented by phytotherapeutics (e.g., standardized preparation of saw palmetto, nettle, Pygeum, pumpkin, rye grass pollen, soy isoflavones, and β-sitosterol) [9,16,17,18,19]. Moreover, researchers are focusing on new herbal alternatives in regard to BPH treatment, specifically traditional Chinese medicine [20,21].

If the patients suffer from moderate or severe stages of BPH, specialists mostly offer drug therapy or surgical possibilities. The medications based on the pharmacological actions of active compounds include α1-adrenoceptor antagonists, 5α-reductase inhibitors, muscarinic receptor antagonists, phosphodiesterase type 5 inhibitors, and vasopressin analogs [10,15].

### 2.1. α1-Adrenoceptor Antagonists and 5α-Reductase Inhibitors

Due to the development and widespread use of medical treatments, surgical options have been pushed into the “second line” of intervention. The most commonly used α1-adrenoceptor antagonists (α1-blockers), and 5α-reductase inhibitors for BPH treatment are summarized in Table 1.

To improve the clinical symptoms of LUTS and to relax the muscle of the prostate gland, α1-blockers can be used. They do not reduce prostate gland enlargement, but they inhibit the binding of noradrenaline to the α1-adrenergic receptors. In this category, alfuzosin, doxazosin, silodosin, tamsulosin, and terazosin are available for BPH treatment with similar clinical efficacy [10]. Among them, silodosin and tamsulosin are subtype-specific antagonists [22]. During their administration, the most frequent adverse events include asthenia, dizziness, and orthostatic hypotension. Tamsulosin showed the highest risk of intraoperative floppy iris syndrome development in connection with cataract surgery. They may have a small beneficial effect on erectile dysfunction, and they do not adversely affect libido. Because of their rapid onset of action, they belong to the first-line drug treatment [10].

Current treatment includes 5α-reductase enzyme inhibitors (finasteride, dutasteride), which block androgen signaling via inhibition of DHT production. After a few months, they could reduce the size of the prostate gland. Their relevant side effects include reduced libido, erectile dysfunction, and ejaculation problems. Since relationships between 5α-reductase enzyme inhibitors and prostate cancer occurrence remained unclear, PSA levels should be monitored during therapy. Due to their slow onset of action, they are only appropriate for long-term medication [10,11].

### 2.2. Other Medicaments and Combination Therapy in BPH Treatment

Medical treatment of BPH includes both static (reduction of prostate growth with 5α -reductase inhibitors) and dynamic components (improvement of smooth muscle contractility with α1-blockers) [22]. Moreover, phosphodiesterase-5 inhibitors (PDE5 inhibitors), muscarinic receptor antagonists, and vasopressin analogs may also have beneficial effects on the BPH-LUTS. Muscarinic receptor antagonists licensed for storage symptoms improvement include darifenacin, fesoterodine, oxybutynin, propiverine, solifenacin, and tolterodine. Because the effects of their long-term administration have not been clarified yet, they should be administered with caution and regular IPSS evaluation. PDE5 inhibitors may also have potential in LUTS improvement, via reducing smooth muscle tone of the detrusor, prostate, and urethra. In Europe, among the licensed PDE5 inhibitors (sildenafil, tadalafil, and vardenafil), only tadalafil (5 mg) has been approved for LUTS therapy. Its administration is contraindicated in patients who use nitrates, potassium channel openers, nicorandil, α1-blockers, or have cardiovascular problems (e.g., unstable angina pectoris, a recent myocardial infarction or stroke, poorly controlled blood pressure, etc.) [10]. In the case of patients with nocturia, desmopressin (vasopressin analog) administration can reduce total urine volume. During its application, regular monitoring of serum sodium levels is essential to prevent hyponatremia [10].

To prevent the disease progression, α1-blocker + 5α-reductase inhibitor combinational therapy may also have potential, especially if the patient accepts long-term medication (Table 1). In cases when detrusor overactivity was demonstrated, co-administration of α1-blockers and muscarinic receptor antagonists showed synergic activity [10,23,24]. In patients with overactive bladder symptoms, β-3 adrenergic agonist (mirabegron) add-on therapy was also investigated as a promising alternative [15]. PDE5 inhibitors in combination with 5α-reductase inhibitors only showed a slight improvement in patients with a larger prostate [25]. However, before the start of these combinational therapies, cost-effectiveness and clinical relevance should be considered.

### 2.3. Surgical Therapy

In cases where the symptoms resist the medical therapy or the disease is in an advanced phase, surgical treatment could be a possible solution. Before surgery, the risk/benefit ratio should be considered, as well as the patient’s full medical history and condition, focusing on the size and shape of the prostate gland. All patients should be informed about the treatment failure, side effects, and possible retreatment options. Most of the procedures are performed via the urethra where the prostate tissue can be removed, compressed, or destroyed by different energy sources [14].

Various forms of surgical treatment for BPH are available today, including conventional techniques, such as transurethral resection of the prostate (TURP), a modified version of this technique, and minimally invasive options (e.g., urethral microwave therapy (TUMT) and laser, electrode, transurethral thermal ablation-assisted techniques) [26]. In comparison with TURP, laser-based methods decrease short-term complications. Similarly, thermal ablation therapies may cause fewer adverse events than conventional techniques, but they can be associated with irritative symptoms and urinary catheterization. A prostatic urethral lift also has potential in the preservation of ejaculatory function; however, it was found less effective in improving urological symptom, in both the short- and long-term [27].

Recently, a new method called convective radiofrequency water vapor thermal therapy, creating necrotic tissue in the prostate, became available. In LUTS treatment, this targeted, controlled water vapor energy applying technique could improve the life quality of patients. However, the effectiveness of this method, in comparison with other techniques, remained unclear [28].

## 3. Medicinal Plants Widely Used in the Treatment of BPH

### 3.1. Cucurbita pepo

Pumpkin (*Cucurbita pepo* L.) belongs to the Cucurbitaceae family, with several varieties grown throughout the world [29]. *C. pepo* is native to South–Central America [30], and has a long tradition of cultivation from Mexico to Argentina and Chile, but it can be successfully grown in Europe, Asia (India and China), and in Western America [31]. Pumpkin is a creeping or climbing annual plant. The oily seeds are ovate-elliptical, flattened, 15–25 × 7–12 mm, and a dark brown to black or creamy white color [32]. Pumpkin seed oil has been used since the end of the 19th century to treat urinary tract problems [33].

Pumpkin contains various biologically active components, such as polysaccharides, para-aminobenzoic acid, sterols, proteins, peptides [34], carotenoids, and γ-aminobutyric acid [35]. Pumpkin seeds have high protein and essential fatty acid content [36], the most important fatty acids being linoleic acid, palmitic acid, stearic acid, oleic acid. Furthermore, it contains non-essential amino acids, e.g., cucurbitin, as well as Δ^5^-, Δ^7^-, and Δ^8^-phytosterols, e.g., sitosterol and stigmasterol [4,30,37]. In addition, pumpkin seeds contain microelements (Na, K, Cr), tocopherol (vitamin E) [38,39], pigments, pyrazine, triterpenoids (e.g., saponins), and phenolic compounds, such as coumarins, and flavonoids [40,41,42,43] (Table 2). 

#### 3.1.1. Preclinical Studies

Pumpkin seed extract was reported to have antitumor [44], hepatoprotective [45], wound healing, anti-arthritis, hair-growth stimulating [46], anthelmintic [47], and antioxidant effects [48]. Moreover, therapeutic activities of pumpkin seed extract include the relief of symptoms associated with prostate disorders [49,50,51], urinary bladder complications [52,53,54], and lower urinary tract diseases [55]. Pumpkin seed extracts can block the increase of prostate weight and protein synthesis induced by testosterone/prazosin [56], inhibiting testosterone-induced hypertrophy [50]; 10 g/day pumpkin extract exerts tonic effects on the bladder and urethra [16,57]. 

In Europe, pumpkin seed oil has been used in folk medicine for treatment of BPH [58]. Efficiency can be achieved alone or in combination with saw palmetto when used to treat BPH [59]. Pumpkin seed oil has been found to reduce testosterone-induced prostatic hyperplasia in rats. In the experiment, pumpkin seed oil was orally administered to rats at 2 and 4 mg/100 g of body weight for 20 days, and both concentrations decreased the prostate size ratio [50]. The mechanism of action of pumpkin seed oil is supposed to involve 5α-reductase inhibition [50]. Some studies also found that pumpkin seeds are rich in zinc, and these elements may help shrink an enlarged prostate [16,60] (Table 2).

#### 3.1.2. Clinical Studies

A multicenter clinical trial in 1998 showed that the symptoms of BPH were relieved after taking capsules with pumpkin seed extract (500 mg) [61]. In a randomized, placebo-controlled, parallel-group trial, which enrolled 1431 men (of average 65 years old) with BPH, the treated group used pumpkin seed extract for 12 months. The treatment showed that the symptoms of BPH were relieved compared to the placebo group [62]. In a study by Leibbrandt and Coulson, pumpkin seed extract was used for 12 weeks by BPH patients in a randomized, double blind, placebo-controlled clinical trial that included 57 males aged 40–80 years. As a result, the pumpkin seed extract reduced the symptoms of treated group compared to the control group [63]. Leibbrandt et al. reported that the IPSS was reduced by 30% in the group treated with oil-free hydroethanolic pumpkin seed extract [64]. In a study involving 100 BPH patients, the symptoms of BPH were relieved after using pumpkin seed oil for 24 weeks [65]. Hong et al. [66] conducted a clinical trial on 47 men with BPH symptoms. Patients received a mixture of pumpkin seed and saw palmetto oil, which significantly reduced IPSS within 3 months. In a clinical trial, the effects of pumpkin seed oil were evaluated in over 2000 men suffering from BPH. The patients used 500–1000 mg/day of the oil for 12 weeks. As a result, the treatment decreased the IPSS by 41.4% and more than 96% of the patients had no undesired side effects, indicating that pumpkin seed oil significantly improved the urinary dysfunction in patients [67].

Pumpkin seeds are extremely safe, but their use may lead to minor stomach upsets, and can be responsible for indigestion, diarrhea [57], and electrolyte loss (due to its diuretic properties) [68]. For these reasons, pumpkin seed oil is contraindicated in the case of concomitant anticoagulant therapies [30]. The use in children and adolescents under 18 years of age, and in pregnant women, is not recommended, because LUTS—in these populations—require medical supervision [69].

### 3.2. Epilobium parviflorum and E. angustifolium

*Epilobium* species, commonly known as willowherbs or fireweeds, are perennial herbaceous, mostly hemicryptophyte plants, members of the evening primrose family (Onagraceae) [121]. The aerial parts of the plant are used as herbal tea or in combination with other herbal substances in herbal medicines. Based on long-standing use, some *Epilobium* species, including *E. angustifolium* L. and *E. parviflorum* Schreb., can be used for the relief of LUTS of BPH, such as difficulty starting urination or a frequent need to urinate [122]. The European and North American traditional uses include treatment of BPH, prostatitis, bladder, and kidney diseases, and other urinary tract associated problems, while based on American traditions, it can also treat diarrhea and other gastrointestinal diseases, cough, various skin and mucosa diseases, bodily injuries, and pain [70].

Plant materials of the *Epilobium* genus are especially rich in polyphenols including flavonoids (kaempferol, quercetin, and myricetin derivatives), phenolic acids (ellagic, chlorogenic, gallic acid), and tannins (ellagitannins and gallotannins in *E. parviflorum*) but some steroids, triterpenoids, and fatty acids were also isolated from them [70] (Table 2).

#### 3.2.1. Preclinical Studies

*Epilobium* is one of the medicinal plants used against the symptoms of BPH, although its mechanism of action is not completely clarified. Nevertheless, preclinical studies reported anti-inflammatory, antioxidative, anti-proliferative, antimicrobial, analgesic, and antiandrogenic activities of the extract [70,71]. Review articles summarized the biological activities of *E. angustifolium,* including the above-mentioned ones besides cytotoxic, immunomodulatory, photoprotective, anti-ulcer properties, and emphasizing its importance in BPH treatment and prostate cancer prevention [72,73,74]. A recent assessment was also made about the wound healing properties of *Epilobium* species due to its anti-hyaluronidase, anti-collagenase, and antioxidant activities [75]. In general, polyphenols are thought to be responsible for the effects of *Epilobium* extracts. Ellagitannins are metabolized by the gut microbiota resulting in anti-inflammatory urolithins [72,76]. Oenothein B, a macrocyclic ellagitannin, seems to be at least one of the major bioactive compounds of *Epilobium* species due to its antioxidant, anti-inflammatory, enzyme inhibitory, antitumor, antimicrobial, and immunomodulatory activity described in several preclinical studies [77]. Due to its large molecular size and relatively high polarity, the bioavailability of oenothein B is very poor, similarly to other ellagitannins, but in contrast to them, oenothein B is not metabolized by the human gut microbiota, and its metabolic pathway is still unknown [72,123,124]. In addition, recent studies suggest that catechin and epicatechin (flavonoids) can have a major role in prostatitis related effects of *E. angustifolium* due to their COX-2 inhibition [125], while quercetin, myricetin, and myricitrin, in the case of *E. parviflorum* in cancer therapy [126], based on molecular docking analysis.

Orally administered *E. angustifolium* caused a slight increase of CYP2D2 and a significant decrease of CYP3A1 expression [78]. Intraperitoneal injection of *E. hirsutum* extract decreased the CYP2E1 and CYP1A1 expression in rats [79]. Another article discussed the effect of intraperitoneal *E. hirsutum* extract and ellagic acid injection on drug metabolism in rats, where both test substances inhibited hepatic erythromycin *N*-demethylase, benzphetamine *N*-demethylase and 7-benzyloxyresorufin-*O*-debenzylase activity, and decreased CYP2B1, CYP2C6, CYP2D2, and CYP3A1 protein levels [80] (Table 2).

#### 3.2.2. Clinical Studies

Despite the promising preclinical results with *Epilobium* species, human clinical trials connected to BPH are limited. Literature search revealed only one randomized, double blind, placebo-controlled clinical trial related to BPH that was performed solely with *Epilobium* extract, and included 128 adult men. The treated group received 500 mg chemically characterized *E. angustifolium* extract (containing ≥ 15% oenothein B) in the form of hard, gastric-resistant capsules daily for 6 months. The *E. angustifolium* treatment significantly improved the IPPS, the post-void residual (PVR), and the number of urinations during the night, but not the PV or the PSA. Tolerance and safety assessment was also conducted, according to which the *E. angustifolium* food supplement was found to be well tolerated, and did not cause any hepatic or renal toxicity [127]. Other human studies with *E. angustifolium* extract focused on its skin photo-protection and anti-dandruff effect [128,129].

Most clinical studies were performed with combined preparations, containing additional components known for their anti-BPH activity. An herbal preparation containing *Cucurbita pepo*, *E. parviflorum* (equivalent to 500 mg dry herb), lycopene, *Pygeum africanum,* and *Serenoa repens* was examined in a randomized, double blind, placebo-controlled clinical trial, where this herbal medicine significantly improved the symptoms of BPH [63]. Another combined preparation, a food supplement containing water-soluble extracts of *Ononis spinosa, Solidago virga-aurea*, *Phyllanthus niruri*, *Peumus boldus*, and *E. angustifolium* (12.5:12.5:18.7:25.0:31.2, respectively, as dried materials) was also studied with 30 patients, and after 1 month of treatment the extract improved the LUTS, such as maximum flow, and the quality of life assessed by the IPPS questionnaire. Related to this study, reduced cell viability and inhibited release of PGE_2_ and COX-2 gene expression were also observed in vitro in human prostate PC3 cancer cells [130]. 

### 3.3. Hypoxis hemerocallidea

The *Hypoxis* genus (family Hypoxidaceae) covers 103 species registered in “The Plant List” [131]. They can be found in most warm temperate and tropical zones of the world (under different synonyms). The most known (and studied) species is *Hypoxis hemerocallidea* Fisch., C.A. Mey. and Avé-Lall. (syn. *Hypoxis rooperi* T. Moore or *Hypoxis rooperi* var. *forbesii* Baker). However, several other *Hypoxis* species and their synonyms were mentioned in published studies in previous years, due to the fact that many *Hypoxis* species are used indiscriminately in traditional medicine and are sold under the common name “African potato” in herbal shops. The plant possesses an underground part, which is neither a tuber, nor a root, nor a rhizoma, but a corm (“bulbous tuber”), a rounded underground storage organ consisting of a thickened base of a stem covered with leaf scales. The dark brown corm (7–10 cm diameter) is covered with bristly hairs, and is bright yellow when freshly cut and turns brown after some time. It has an unpleasant bitter taste. 

As described earlier [4], the *Hypoxis* corm is traditionally used to treat a wide variety of diseases/conditions (prostate hypertrophy, burns, cancer, cardiac diseases, dizziness, headaches, impotency, intestinal parasites), and to boost the immune system. 

The corm contains (up to 10%, dry weight) a unique secondary metabolite, hypoxoside, its aglycone rooperol, β-sitosterol, stigmasterol, stigmastanol, as well as the newly described hypoxhemerolosides A–F, curcapicycloside, obtuside A, interjectin, crassifoside F, acuminoside, geraniol glycoside, vanillic acid, β-arbutin, orcinol glycoside [81,82] (Table 2).

#### 3.3.1. Preclinical Studies

In addition to previously summarized data (e.g., β-sitosterol related increase of TGF-β1 expression and protein kinase C-α activity in the stromal cells of the human prostate, in vitro anti-inflammatory activity of rooperol, or the possibility of an interaction of *Hypoxis* preparations with CYP450 isoforms 1A2, 2A6, 2B6, 2C8, 2C9, 3A4, and 3A5) [4] (Table 2), there are only three new (and not BHP-related) studies with *Hypoxis* or its constituents. Two of these studies, investigating the outcomes of concomitant administration of various *H. hemerocallidea* extracts with indinavir [132] or a lopinavir/ritonavir combination [133], did not find statistically significant changes in the pharmacokinetics of the antiviral agents.

In the case of streptozotocin-induced diabetes mellitus, improved antioxidant enzyme activities were measured in adult male Wistar rats, both under normal and under oxidative stress conditions, following treatment with 800 mg/kg *H. hemerocallidea.* The results obtained in this study showed that effects were dose independent. The sperm motility and morphology showed the greatest improvement in the diabetic group treated with *H. hemerocallidea* [134].

#### 3.3.2. Clinical Studies

Currently, no clinical trials have been published, directed at the application of *Hypoxis* extracts in the treatment of BPH, which of course does not give the opportunity to make a clear position on the therapeutic efficacy of the use of *Hypoxis* extracts (these only at the level of food supplements, not registered drugs), or to assess the benefit–risk ratio.

### 3.4. Solanum lycopersicum

*Solanum lycopersicum* L. syn. *Lycopersicum esculentum* Mill., or the tomato plant, belongs to the Solanaceae family. It is native to the Andes of South America, from where it has spread to all temperate and tropical regions; it is considered today as one of the world’s most important vegetables. It is an annual plant that grows up to 2 m in height and is cultivated today mainly for its edible fruits [135].

*S. lycopersicum* fruits are typically composed of 94.5% water, 3.89% carbohydrates (1.2% of total dietary fiber and 2.63% of sugars), 0.88% protein and 0.2% total lipid (fat), while other nutrients and phytochemicals include minerals (e.g., 237 mg potassium, 24 mg phosphorus, 11 mg magnesium, 10 mg calcium), vitamins (e.g., 13.7 mg vitamin C, 0.12 mg γ-tocopherol, 0.6 mg niacin), and tetraterpenes (e.g., 449 μg β-carotene, 101 μg α-carotene, and 2570 μg lycopene, collectively known as carotenoids) [83] (Table 2). Lycopene has been widely studied in relation to prostate dysfunctions, including BPH and prostate cancer [136,137,138].

#### 3.4.1. Preclinical Studies

Lycopene is a non-provitamin A carotenoid with antioxidant activity exerted by quenching of singlet oxygen and scavenging peroxyl radicals [84]. Additionally, it was shown to decrease the expression of nicotinamide adenine dinucleotide phosphate oxidase 4, consequently reducing reactive oxygen species generation [85]. Lycopene has also been shown to possess anticancer and anti-inflammatory activities [86] (Table 2). Lycopene can induce cell-to-cell communication and controls cell growth [84]. It antagonized transforming growth factor β-induced metastasis in vitro [85]. In LNCaP human prostate cancer cells, lycopene induced apoptosis, as more cells were in G_2_/M cell cycle phase and less were in S-phase upon treatment with 5 µM lycopene [139]. Another study showed a similar effect of lycopene extract on the induction of apoptosis in primary human prostate cancer cells with an upregulation of p53 and Bax, and downregulation of Bcl-2 [140]. Induction of apoptosis following tomato sauce consumption was seen in dissected tumors of BPH and prostate cancer patients [141]. Tomato and lycopene were also shown to reduce the risk of cardiovascular disease [142]. The underlying mechanism might be the antioxidant activity of lycopene, as it decreased low-density lipoprotein levels and its oxidation [143]. Antithrombotic activity was also observed, as tomato extracts reduced platelet aggregation [144]. The anti-inflammatory activity of tomato and lycopene might also contribute to beneficial effects in cardiovascular health. It was shown to reduce tumor necrosis factor-α concentrations in healthy individuals [145]. Lycopene alleviated chronic prostatitis/chronic pelvic pain syndrome in a rat model, presumably due to its anti-inflammatory properties: cytokines tumor necrosis factor-α, interleukin-1β, interleukin-2, and interleukin-6 were downregulated; phosphorylation of mitogen-activated protein kinase (MAPK) and nuclear factor-κB (NF-κB) decreased, while phosphorylation of nuclear factor erythroid 2-related factor 2 (Nrf2) increased [146]. In normal prostate tissue in rats, lycopene also reduced androgen signaling, insulin-like growth factor 1 (IGF-1) expression and proinflammatory cytokines [147].

#### 3.4.2. Clinical Studies

Lycopene may be beneficial in the prevention of prostate cancer [148]. Patients with BPH are at a higher risk of developing prostate cancer [149], but few clinical studies addressed the use of tomato or derived products in BPH. In a recent phase II clinical study, tomato-based food supplement improved lower urinary tract problems in patients with BPH [150]. Patients receiving 5 g of the supplement daily for 2 months reported no adverse effects and fewer lower urinary tract problems, leading to a statistically significant improvement of the quality of life. On a molecular level, a reduction in PSA was observed, but was only significant in patients who had higher (above 10 ng/mL) baseline concentrations of PSA. Approximately 11% decrease in PSA concentration upon ingestion of 10 g of tomato paste for 10 weeks was observed in BPH patients in another study [151]. A defined dose of lycopene, i.e., 15 mg daily taken for 6 months was given to patients with histologically determined BPH and absence of prostate cancer in a study by Schwarz et al. [152]. Lycopene-treated patients had a statistically significant reduction in PSA levels as compared with placebo group and experienced an improvement of symptoms. No prostate enlargement occurred in lycopene-treated patients as opposed to the placebo group, where the prostate enlargement was statistically significant at the end of the 6-month study.

Excessive consumption of tomato-based products can have harmful effects, as it can lead to gastroesophageal reflux disease (due to the presence of organic acids), kidney problems (due to high potassium and oxalate concentrations), irritable bowel syndrome (due to high tomato skin and seeds consumption), lycopenodermia (orange skin discoloration due to high lycopene blood levels), urinary problems (due to the presence of organic acids), body aches, and arthritis (in case of consumption of unripe green fruit containing higher concentrations of toxic steroidal glycoalkaloids tomatine and solanine) [86]. A combination of lycopene and alcohol induced CYP 2E1 expression and inflammation in rats, but clinical importance of this has not yet been shown [153]. Tomato is a known allergen in some individuals and can lead to anaphylaxis [154,155].

The beneficial effects of tomato and derived products in BPH are mainly attributed to the biologically active carotenoid compound lycopene that has antioxidant, anti-inflammatory, and anticancer activities. The use of tomato-based or lycopene supplements was efficient in alleviating the symptoms of BPH in several clinical studies. However, adverse effects are possible; therefore, more research is needed to determine safe and efficient dosage regimen of tomato and derived products in BPH.

### 3.5. Pinus pinaster

French maritime pine bark extract exhibits antioxidant and anti-inflammatory effects and has been studied in a variety of clinical conditions, including asthma, attention deficit hyperactivity disorder (ADHD), chronic venous insufficiency, cardiovascular disease, diabetes, and erectile dysfunction. However, the publication of many methodologically weak clinical studies makes it difficult to provide clear support for the use of pine bark extract for each condition [156]. The pine bark is harvested, powdered, and typically extracted via a patented methodology to produce Pycnogenol^®^ (Horphag Research, Geneva, Switzerland) [157] and is therefore a patented preparation of *P. pinaster* standardized to 70 ± 5% procyanidins in compliance with the United States Pharmacopeia [87]. Procyanidins are biopolymers of catechin and epicatechin subunits, important components of human nutrition, with studies increasingly suggesting that this standardized extract has favorable pharmacological properties [158]. Importantly, procyanidins are considered powerful antioxidants found in significant quantities in foods including grapes, berries, and red wine, and are marketed widely through various different health promotional products for various chronic disorders [88]. Additional important constituents include taxifolin, cinnamic, ferulic, caffeic, and benzoic acid (Table 2). Pycnogenol^®^ has been shown to have beneficial effects in a variety of chronic diseases, including obesity, metabolic syndrome, and type 2 diabetes mellitus, alongside associated dyslipidemia and hypertension [87]. Evidence further suggests beneficial effect in UV-induced radiation damage, asthma, and systemic lupus erythematosus, alongside improvements in osteoarthritis, cognitive function, specifically attention deficit disorder (ADD) [157,158,159]. Pleiotropic effects, including a beneficial effect in BPH, are reported by Rohdewald et al. [160].

Pycnogenol^®^ is considered safe with low toxicity in acute or chronic exposures [158]. The dosages currently recommended through clinical trials range from 100 to 360 mg per day. Doses of pine bark extract have been studied in clinical trials, most commonly at 150 mg per day in three divided doses [156].

#### 3.5.1. Preclinical Studies

French maritime pine bark protects against oxidative stress by increasing the intracellular synthesis of antioxidant enzymes and by acting as a potent free radical scavenger via the regeneration and protection of vitamins C and E. Anti-inflammatory activity has been demonstrated in vitro and in vivo in animals, including protection against UV radiation-induced erythema. Immunomodulation has been observed in animal models and in patients with lupus. French maritime pine bark counteracts adrenaline and noradrenaline-induced vasoconstriction by increasing endothelial nitric oxide synthase (eNOS) activity and preventing smoking-induced platelet aggregation. It has been shown to relieve some premenstrual symptoms, including abdominal pain. This effect may be related to the spasmolytic action of certain phenolic acids [89,159]. It has many other pharmacological activities, including protection against ovariectomy-induced bone loss in rats [90].

There are generally few studies for Pycnogenol^®^ in male reproduction, especially in preclinical studies. These suggest a beneficial effect on sperm quality. However, there is more substantial evidence for the use of Pycnogenol^®^ in erectile dysfunction. In a rat model, where spermatotoxicity was induced by administration of 30 mg/kg/day α-chlorohydrin for 7 days, co-administration of Pycnogenol^®^ (20 mg/kg/day) showed reduction in malondialdehyde, increased glutathione, catalase and peroxidase levels in the epididymis, improved histopathological changes, reduced apoptosis and resulted in improvement in sperm motility, compared to the control [161].

A study investigating whether Pycnogenol^®^ could prevent BPH induced by testosterone propionate (TP) was conducted in rats. One group was used as normal control rats and the other groups received subcutaneous injections of TP for 4 weeks to induce BPH. In the two treatment groups, Pycnogenol^®^ (20 or 40 mg/kg) was administered daily for 4 weeks by oral gavage, concurrently with the induction of TP. Results indicated that Pycnogenol^®^ inhibits the development of BPH and that this is closely associated with a reduction in DHT levels. [91] (Table 2).

#### 3.5.2. Clinical Studies

A nonrandomized clinical trial studied 19 subfertile men who received 200 mg/day of Pycnogenol^®^ for a period of 3 months. Semen analysis showed an improvement in capacitation (38%) and mannose receptor binding capacity (19%) compared to baseline (before treatment), suggesting that this treatment may improve natural or induced reproductive outcomes in cases of teratozoospermia. Prelox^®^R is a combination of Pycnogenol^®^, alongside L-arginine, L-citrulline, and roburin. In a double-blind, randomized, controlled crossover study, subfertile men (*n* = 50) underwent monthly sperm analysis in a pretreatment phase (1 month), followed by a treatment or placebo phase (1 month), then a washout phase (1 month), followed by the crossover phase (1 month). The study showed a significant improvement in sperm volume, concentration, motility, viability, and morphology compared to the placebo treatment, with an increase in intracellular eNOS activity in spermatozoa [92].

Prelox^®^R demonstrated also improvement in erectile function in a male cohort (*n* = 50) over a 1-month treatment period in a randomized, placebo-controlled, double-blind crossover study using the International Index of Erectile Function as the primary outcome. Significant improvements in erectile function were reported compared with placebo [162]. This was consistent with previous studies reporting that Prelox^®^R restored normal erectile dysfunction over 1-month treatment, alongside significant improvements in sexual intercourse frequency (doubled) and increases in serum testosterone and sperm eNOS [163,164]. Taken together, these studies suggest some consistency for the effect of Pycnogenol on erectile dysfunction, however, they are combined with L-arginine; therefore, the effect of Pycnogenol^®^ alone is not clear.

Another study evaluated the efficacy of Pycnogenol^®^ supplementation in terms of safety and tolerability for preclinical or borderline early symptoms of BPH in otherwise healthy subjects over a 60-day period. Seventy-five healthy men with symptoms and signs of incipient BPH were included. Subjects were divided into three groups: (1) control group using standard management (SM) without surgical indications, based on the avoidance of anticholinergic, sympathomimetics, opioids drugs; patients were instructed to void regularly, avoid long seating periods, exercise regularly, hydrate appropriately, preferably avoiding caffeine and spices, follow a low-sugar and low-salt diet; (2) A group using SM plus Pycnogenol^®^ 150 mg/day; (3) A group using standard pharmacological management, including dutasteride (one capsule, 0.5 mg/day) and/or finasteride 5 mg/day. 

The results showed BPH symptoms like emptying, frequency, intermittency, urgency, weak flow, straining, nocturia, were all significantly improved with Pycnogenol^®^ (*p* < 0.05) and the difference with both control groups was statistically significant (*p* < 0.05). So Pycnogenol^®^ may be an important option for self-management of BPH in otherwise healthy men [165].

In conclusion, relatively few preclinical or clinical studies have been conducted with *P. pinaster* extracts in andrology [166], and the limited studies suggest a role in protecting against induced spermatotoxicity through improved oxidative stress markers. It is also suggested to be useful in teratozoospermia patients. In combination with L-arginine, L-citrulline, and roburin (Prelox^®^R), Pycnogenol^®^ may improve fertility outcomes in subfertile males. However, the most significant clinical indication of this combination has been demonstrated in erectile dysfunction. This appears to be independent of the influence of the amino acid L-arginine, and it is mediated by vasodilation. There are few studies, to date, on the use of Pycnogenol^®^ in subfertility, but the evidence is good for the use of Prelox^®^R in erectile dysfunction. Based on the current evidence, further studies are warranted for the use of Pycnogenol^®^ in andrology, particularly in oxidative stress-induced infertility, erectile dysfunction, and as recent studies show in the treatment of BHP.

### 3.6. Roystonea regia

*Roystonea* is a genus of the Arecaceae family, which contains ten species, the most famous of which is *Roystonea regia* (Kunth) O.F. Cook, known also as Cuban Royal Palm [167]. It grows from south Florida through Central America and some Caribbean islands to South America. This majestic plant grows from 15 to 21 m tall, with a canopy spread of up to 7.5 m in diameter. The dark purple or black fruits are smooth, ovate, and measure 13 mm in length. The fruit is a rich source of oil [168].

Basic information related to the chemistry of the D-004 extract (a lipid extract of *Roystonea* fruit, containing oleic, lauric, palmitic and myristic acids) was given in an earlier review [4], as well as preclinical studies on this extract (antioxidant effects in prostate tissue, competitively inhibiting the prostatic 5α-reductase, and the sympathetic-induced contraction of the smooth muscle in rat-isolated deferens tube), as well as in vivo studies on prevention and improving BPH induced by testosterone in rats, and a randomized, double-blind study on healthy volunteers, indicating significant antioxidant effects on plasmatic oxidative markers of D-004 taken for 6 weeks (Table 2). However, some BPH therapy-related articles were omitted in [4]; thus, they will be presented here, together with recent articles.

#### 3.6.1. Preclinical Studies

Arruzazabala et al. investigated the effects of D-004 on phenylephrine-induced contractions in isolated rat prostate strips. D-004 significantly and dose-dependently inhibited the contractions through a noncompetitive mechanism [169] (Table 2).

D-004 extract was evaluated in a subchronic (8 weeks) study in mice. No evidence of treatment-related toxicity was detected. Thus, body weight gain, clinical observations, food consumption, blood biochemical, hematology, organ-weight ratios, and histopathological findings were similar in the control and treated groups. This study supports that D-004 orally administered up to 2000 mg/kg/day did not induce treatment-related toxicity [93]. The same research group investigated the long-term oral toxicity of D-004 in rats. No clinical signs of toxicity were observed throughout the study. Thus, 12 months of oral treatment of rats with D-004 (up to 2000 mg/kg/day) did not show evidence of its toxicity [170]. Finally, Gutiérrez et al. demonstrated that oral administration of D-004 (up to 1500 mg/kg/day) for 24 months was devoid of long-term oral toxicity and/or carcinogenicity in male and female rats, and that the highest dose tested was the NOAEL [171].

Arrebolal et al. (2009) studied a risk of changes in the appearance frequency of micronuclei during oral administration of repeated doses of D-004 in bone marrow male OF-1 mice for 8 weeks. There were no deaths or clinical signs of toxicity or significant differences among controls and treated ones regarding the frequency of micronucleated polychromatophil erythrocytes, and the cytotoxic index. D-004 administered p.o. has neither clastogenic nor cytotoxic activity in vivo [172]. 

Thus, these mid- and long-term animal experiments indicated with a high degree of certainty that any therapy with D-004-containing products should be non-toxic.

#### 3.6.2. Clinical Studies

The aim of a single recent study was to compare the efficacy and tolerability of D-004 with terazosin on LUTS in 100 men (at least 50 years of age) with BPH in an open, randomized, comparative design. D-004 (320 mg/day) and terazosin (5 mg/day) significantly reduced the IPSS at the end of the 6 months of therapy, by 74.2% and 66.1%, respectively. Although the average size of the prostate was reduced in both groups, this reduction reached statistical significance only for D-004. Both treatments were safe, while D-004 was better tolerated than terazosin [173].

### 3.7. Prunus africana

The African cherry or African plum or African prune tree (*Prunus africana* (Hook. f.) Kalkman) (syn. *Pygeum africanum* Hook. f.) belongs to the Rosaceae family. The tree occurs in the mountain regions above 1500 m above sea level in Southern and Central Africa [174], as on the islands of Grande Comore, Bioko, and São-Tomé [100], and in Madagascar, Comoros Islands, and the Gulf of Guinea [4]. It is an evergreen tree, which grows up to 30–40 m height developing a spreading canopy [94].

The powdered bark has been used by indigenous people for urinary tract disorders and as an aphrodisiac drug in the tropical and subtropical regions of Africa [174,175]. The bark is traditionally applied in the treatment of cough and cold in Kenya [176] and South Africa [177]; asthma [178], malaria and prostate cancer in Kenya [179]; BPH in Ethiopia [180] and Mozambique [181]; as anticancer drug in Uganda [182]; for jaundice in Ethiopia [183]; for tuberculosis, HIV, and stomach problems in South Africa [177]; for mental disorders, diabetes, skin infection, ulcers, gonorrhea, as well as for hypertension,; while the root and the fruit for chest pain; the leaf for fever, and e.g., for mental disorders, diabetes, skin infection, ulcers, gonorrhea, and hypertension [184,185,186,187,188].

The effectivity of the peeled bark in the treatment of BPH [189] was detected only in the 1960s [190], which was followed by an intensive bark harvesting in Cameroon in the 1970s [94]. The drug was imported to the European market from French colonies. The bark became a popular and effective drug in Western medicine, followed by even more intensive and inappropriate harvesting methods, which caused an overexploitation of the tree. Numerous international programs were launched to rescue the species, and to supply the medicinal and pharmaceutical demands. The products of the tree are available mostly in French medicine and in the USA [174].

The drug part of African plum is the dark brown bark [100], which can be peeled by various tools from the trunk of the tree [94]. The dried bark can be prepared with organic solvents or supercritical extraction in remedies [174].

The bark contains phytosterols, e.g., β-sitostenone [95], β-sitosterol, and its derivatives [94], including esters and glucosides, e.g., β-sitosterol-3-*O*-glucoside [96], atraric acid [96], and ester derivatives of long-chain alcohols, such as n-docosyl-*trans*-ferulate and n-tetracosyl-*trans*-ferulate. In addition, fatty acids (mostly palmitic acid) [100], triterpenes (e.g., oleanolic acid, ursolic acid, 24-O-*trans-*feruloyl-2α,3α-dihydroxy-urs-12-en-28-oic acid) [97], lauric acid, myristic acid, ferulic acid and its esters [191], atranorin, cholesterol, N-butylbenzene sulfonamide [192], proanthocyanidins [113], hydroxybenzoic, linoleic, stearic, arachidonic, behenic, and lignoceric acids [113] were also isolated from the bark extracts, while α-amyrin, phytol, vanillin, benzenedicarboxylic acid, squalene, nicotinic acid, campesterol, stigmasterol, and α-tocopherol were identified in the leaves of the species [96] (Table 2).

#### 3.7.1. Preclinical Studies

The anti-inflammatory effects of the bark [99] contribute to the decrease of the obstructive symptoms of BPH [174]. The effects for prostate hypertrophy were documented in several pharmacological studies, e.g., on mice and rats [57,102,193,194,195,196]. The bark inhibits the basal growth of prostate stromal cells in rats, stimulated by EGF, IGF-I, bFGF (direct activators of protein kinase C), TPA (tissue plasminogen activator), and PDBu (phorbol 12,13-dibutyrat) [196]. 

The inhibitory potential of lipoxygenase was proved in various in vitro studies. The antiproliferative potential of the extracts plays a role to slow or stop the process of hyperplasia and urinary dysfunctions [102,174,196,197,198,199]. This effect has been observed in rats, using Tadenan^®^, which is an extract of the plant, before and after administration of DHT [200]. This extract prevents the activation of free radicals and metabolizing enzymes, protects the intracellular membrane against the harmful effect of free radicals, and decreases the dysfunction of the urinary bladder [98]. The use with DHT decreased the frequency of micturition as pretreatment in rats, but by itself, it increased the rate of urine production and the volume of micturition. In addition, the weight of the prostate increased in the group treated by the extract and DHT, but it decreased in the animals treated only by the extract. These results also highlight the reducing potential of the extract against the harmful effects of DHT on micturition [201]. 

In another experiment, primary and organotypic cultures of human prostatic stromal myofibroblast cell line WPMY and prostatic epithelial cell line PNT2 were investigated: the oral intake of the extract resulted in serum levels of active compounds, which inhibited the proliferation of cultured myofibroblasts prostatic cells [202].

The extracts also inhibit the activity of 5α-reductase [174,203], DHT and estrogen receptors [4], the progesterone and androgen receptors [97]. In addition, its α-adrenergic antagonism potential was also described [4] (Table 2).

In animal models, the extracts decreased the stimulus sensitivity in the urinary bladder, which enhanced the irritative symptoms [174]. In addition, the bark extract regenerates the secretion of the prostate epithelium, and possesses anti-inflammatory activity through the inhibition of 5-lipoxygenase [101].

Cytotoxic activity of the drug was investigated on HLaC79 cells and mucosal keratinocytes, where the extracts increased the apoptotic cell fractions [204].

The mechanisms of the effect of the bark are definitely not clarified yet, and the compounds, which can be responsible for the medicinal effect, were not identified with certainty [174].

#### 3.7.2. Clinical Studies

The activity of the bark extract related to prostate hypertrophy was described in numerous studies [58,175,189,205,206]; however, double blind placebo-controlled trials produced variable results, and inadequate reports are available on the safety and efficacy of the extracts [68,207]. The validation and mechanism of action are poorly defined, possibly including growth factor inhibition, anti-inflammation, and antiandrogenic action of the drug [208], and only a few reviewed studies are available [209].

According to the endorsed monograph in the European Union herbal monograph of the European Medicines Agency (EMA), the plant extracts provided a large improvement in a combined outcome of urologic symptoms with BPH [209]. The clinical benefits of the plant in BPH patients are due to the multiple mechanisms, which include inflammatory, hormonal, and bladder components [210]. 

An earlier study reported the effect of the bark extract in the case of nocturia and incomplete bladder emptying after 6 weeks therapy, compared to placebo group [211], and the improvement in micturition [212]. In another study, 750 men were involved in a randomized, double blind, placebo-controlled trial, treated twice daily by Tadenan^®^ [213,214]. Some randomized clinical trials confirmed its significant efficacy in improving urinary symptoms [4,175], compared to placebo control, such as influencing, e.g., the rate of the urine flow, the urine volume, the urination frequency, and the nocturia in beneficial way [174,208]. The plant extracts were found to be efficient in patients with BPH at doses of 50 mg twice daily and 100 mg once daily, in a 2-month randomized, double-blind study, which was confirmed by further improvement after 12 months [215].

Various remedies of the plant are also available in many countries showing an increased prescription index globally [216]. Tadenan^®^ is recently used in France, Germany, and Austria [175]. According to some studies and traded herbal remedies of the drug, the most frequently recommended dose is 100–200 mg/day for 1–2 months [174]. There are no available reports on the overdosage [209].

Further clinical studies are required, e.g., on standardized extracts and for validated methods for more data on the established medicinal and pharmaceutical use of the bark of African plum.

According to recent references, no interactions are known among the remedies of the plant and pharmaceutical products. Only some side effects, such as diarrhea, constipation [174], and mild gastrointestinal problems can be mentioned for its use [19,217]. There are no available tests on reproductive toxicity and carcinogenicity of the extracts [209].

### 3.8. Secale cereale

*Secale cereale* L., commonly known as rye, is a grass (family Poaceae) originating from Turkey. It grows up to 1.5 m height, with a typical spike inflorescence. It is extensively cultivated today throughout the world, particularly in regions with temperate climate, as a food crop [218]. In terms of treatment of BPH, the medicinal part of the plant is the pollen.

A detailed chemical profile of *S. cereale* pollen is, to the best of our knowledge, not available in scientific literature. Pollen is typically composed of carbohydrates, amino acids, proteins, phenolic compounds, sterols, triglycerides, plant pigments, such as carotenoids and flavonoids, minerals and sporopollenin [103,104,105,218] (Table 2). A standardized mixture of pollen of *S. cereale* (92%), *Phleum pratense* (timothy; 5%) and *Zea mays* (corn; 3%) branded as Graminex [106] has been studied by Locatelli et al. [219], and carvacrol, a monoterpenoid, was identified as the predominant phenolic compound, followed by polyphenols quercetin, rutin, chlorogenic acid and gallic acid [219]. The microbiologically digested extract of the pollen mixture branded as Cernilton^®^ contains a large portion (40%) of *S. cereale* pollen, in addition to timothy (26%), maize (26%), pine (5%), orchard grass (2%), and alder (1%) pollen, as specified by one of the manufacturers [220,221]. Cernilton^®^ is a tablet formulation prepared from the cernitin pollen extract, which consists of water-soluble and acetone-soluble fractions, and is known to contain hydroxamic acid and β-sterols [220,222].

#### 3.8.1. Preclinical Studies

Based on early toxicologic and clinical studies, in 1994, Commission E approved the use of *S. cereale*, *P. pratense,* and *Z. mays* pollen extract for treatment of micturition difficulties associated with Alken stage I–II benign prostate enlargement [106,223]. Cernilton^®^ has been used to treat BPH for nearly 40 years [220]. The mechanism of action of rye grass pollen is not yet well known. Hydroxamic acid, contained in Cernilton^®^, was found to inhibit the growth of DU-145 prostate cancer cells, but showed little selectivity for prostate cancer cells, as it inhibited the growth of other cancerous cell lines as well [224]. This was not the case with the effect of the water-soluble fraction of Cernilton^®^, which selectively inhibited the growth of cells derived from the human prostate [225]. Cernilton^®^ was also associated with inhibition of cyclooxygenase and the 5-lipoxygenase activity in rat basophilic leukemia cells, indicating its mechanism of action involves the inhibition of prostaglandin and leukotriene synthesis [226]. In a mouse bladder muscle model, cernitin pollen extract, its water-soluble fraction, and its acetone-soluble fraction induced contractions [107]. In a rat urethral smooth muscle, inhibition of contractions was observed with cernitin pollen extract and both fractions [227]. In an aged and castrated Wistar rat model of sex-hormone induced prostatitis, it had a recovery action [228]. The effect was partially attributed to its anti-inflammatory activity, as cernitin pollen extract reversed increased levels of TNF-α and IL-6 cytokines in this in vivo model. In an in vitro study on human prostatic cell lines and peripheral blood mononuclear cells upregulation of anti-inflammatory, as well as proinflammatory cytokines was observed [229]. Additionally, androgen receptor and PSA expression were decreased in a WPMY-1 human prostate cell line. Cernilton’s anti-inflammatory activity was addressed in a study in patients suffering from chronic prostatitis or prostatodynia, where Cernilton^®^ was found to decrease leucocyte count in the urine and complement C3/ceruloplasmin levels in the ejaculate [108] (Table 2).

#### 3.8.2. Clinical Studies

Similar to in vitro studies elucidating the mechanism of action of *S. cereale*, the majority of clinical data is available on Cernilton^®^ or other pollen mixture extracts, as opposed to pure *S. cereale* extract. Clinical studies in humans confirmed that mixtures containing *S. cereale* pollen extracts were efficient in the treatment of BPH. A study by Yasumoto et al. included 79 patients, who were given 126 mg of cernitin pollen extract for 12 weeks [109]. The results were compared to pre-treatment parameters in these patients. The maximum and average urine flow rates were increased, while residual urine volume was decreased. No changes were observed in PV upon 12 weeks of the treatment, but a decrease in PV was present in subjects who continued the treatment for 1 year. Altogether, the magnitude of the effect of *S. cereale* in BPH was estimated as mildly beneficial. In a double-blind and placebo-controlled study by Buck et al., residual urine volume and anteroposterior prostate diameter upon treatment with Cernilton^®^ decreased, while no changes in flow rate were observed [223]. The authors concluded that Cernilton^®^ might be beneficial in patients with mild to moderate symptoms of BPH. Similar conclusions were drawn in a systemic review of clinical studies on the use of Cernilton^®^ for BPH by MacDonald et al. in 2000 [224]. While the limitations of the available data from clinical studies were pointed out (e.g., short study duration, lack of control groups, limited number of included subjects, insufficient data on the quality of the used extract), Cernilton^®^ was found to modestly improve subjective LUTS, such as nocturia in BPH; however, the objectively measured mean urine flow rate was not improved as compared with placebo treatment. In a study published after the systemic review, BPH patients were treated with 375 mg or 750 mg Cernilton^®^ daily for 4 years [230]. Both dosage regimens showed an improvement in BPH, but the higher dose was found to be more effective, with no adverse events noted. PSA was monitored in this study and no statistically significant differences were observed in its levels. In a more recent study, Cernilton^®^ improved LUTS and sexual function in patients with BPH with moderate to severe prostatitis [231]. Still, more clinical studies are needed on Cernilton^®^ for BPH, as exemplified by the withdrawal of the 2011 update of the systemic review by MacDonald et al., which was published in 2000, due to the lack of adequate data [232,233]. 

The systemic review by MacDonald et al. concluded that Cernilton is well-tolerated [222]. A single case of an adverse effect was reported, and was manifested as mild nausea [222,223]. However, caution should be applied due to a possibility of allergic reactions to ingested pollen [57,234].

In conclusion, *S. cereale* pollen extracts are moderately effective for the treatment of BPH. Patients report mild to moderate improvement of LUTS. However, most data available are on a pollen extract containing a mixture of pollen types, with *S. cereale* constituting just a part of the supplement. More studies with defined dosage of *S. cereale* pollen extract in a long-term treatment and with appropriate controls are needed to fully elucidate the effectiveness of *S. cereale* in BPH.

### 3.9. Serenoa repens

The genus *Serenoa* contains a single species *Serenoa repens* (W. Bartram) Small, known as saw palmetto, with ten botanical synonyms, the most popular being *Sabal serrulata* (Michx.) Schult. f. [235]. It grows in southern coastal regions of USA, and tropical Middle and South-America. The plant is a small (up to 2 m) perennial bush with 1 m wide leaves having 15 to 30 sharp-ending segments. The fruit is an ovoid or subspherical, dark brown or blackish drupe, one-seeded, up to 2.5 cm long and 1.5 cm in diameter [236]. The European Pharmacopoeia requires minimum 11.0% of total fatty acids in the dried ripe fruit of *S. repens* [237].

Based on a large amount of verified scientific data, the drug Sabalis serrulatae fructus was classified as a starting material for herbal medicines in the category of well-established use for the symptomatic treatment of BPH, ATC code: G04CX0, and as a traditional medicinal product for the relief of LUTS related to BPH, after serious conditions have been excluded by a doctor [110]. Therefore, this article only presents relevant and more recent data that are not part of the related assessment report [238] (Table 2).

#### Clinical Studies

In a randomized, double blind trial, the effect of a hexanic extract from *S. repens* (320 mg per day for 6 months) on prostatic inflammation was investigated in patients, using specific histological and immunohistochemical criteria. The results showed that treatment with this extract resulted in significant decrease in all scores (histologic grading, aggressiveness grading, and total score) at the second biopsy. The difference in inflammation improvement was significant for the extract group when compared with the control group [111]. 

In a recent randomized, double-blind, placebo-controlled study, 44 Japanese men aged 40–69 years who were experiencing urination issues, such as urinary urgency, increased urinary frequency, urinary incontinence, and awaken ≥2 times at night to urinate were enrolled. The intervention period was 12 weeks. Capsules tested contained either 320 mg of *S. repens* fruit supercritical fluid extract or placebo. The extract group showed a significant decrease in subjective symptoms related to urination issues. No adverse effects were observed [112]. 

The systematic review and meta-analysis to compare *S. repens* with tamsulosin in the treatment of BPH after at least 6-month treatment cycle was done by Cai et al. Four studies involving 1080 patients (543 in the *S. repens* group and 537 in the tamsulosin group) were included in the meta-analysis. The results were as follows: compared with tamsulosin, *S. repens* had the same effect in treating BPH in terms of IPSS. The incidence of adverse reactions was similar for *S. repens* and tamsulosin, such as rhinitis, fatigue, dizziness, postural hypotension, dry mouth, and headache. For side effects, *S. repens* was well tolerated compared with tamsulosin, especially in ejaculation disorders [239].

Different conclusions were made in the very recent network meta-analysis of 2115 articles. Authors demonstrated that in a short-term follow up, no clinically meaningful improvement in IPSS of (non)hexanic lipidosterolic extract of *S*. *repens* has been demonstrated over placebo or α-blockers. On the contrary, they could demonstrate a long-term (12 months) benefit of these extracts in the treatment of men with LUTS secondary to benign prostatic enlargement. Hexanic extracts showed a greater improvement than non-hexanic ones. Overall, both hexanic and non-hexanic extracts showed a lower value of rank compared with all α-blockers. However, for patients who want to avoid side effects of α-blockers and do not need rapid efficacy, *S*. *repens* extracts could have a rationale [240].

Interpretation of recent clinical studies or a meta-analysis of all published trials does not provide unambiguous conclusions regarding the efficiency of *Serenoa repens* medicines. The magnitude of effects of hexanic extracts and non-hexanic ones, in terms of clinically meaningful improvement, was similar to the placebo for IPSS. One may speculate that the mechanism of action of extracts needs much more time (more than one year), and extract action is related to the quality of the plant source as well as to the method of preparation (extraction). Finally, side effects of *Serenoa* extracts treatment are variable because of the above-mentioned extract quality.

### 3.10. Urtica dioica

*Urtica dioica* L. (Urticaceae family), is known under the common names stinging nettle, common nettle, and Ortiga. It is a perennial herbaceous plant native to Eurasia, which grows on damp soils, meadows, and abandoned fields in dappled-shaded spots [241]. The root extract has been used traditionally for the treatment of symptomatic BPH [68]. The fresh and dried flower parts are traditionally used for joint pain and urinary tract infections, as well. Moreover, it can be used externally as a remedy for hair loss, against seborrhea and dandruff of the scalp [30]. Moreover, it is used to treat diarrhea, acne, and diabetes, and to improve circulation and low blood pressure [4,114].

The most important active compounds of the leaves are sterols (β-sitosterol, hydroxy-sitosterol) and flavonoids (rutin, kaempferol, quercetin). Moreover, the leaves contain minerals (calcium, potassium), tannins, acids (salicylic, malic acids, caffeic acid), and amines (histamine) [4,98]. The roots of stinging nettle contain a mixture of water-soluble compounds, including lectins (mixture of isolectins from 0.2 to 0.6%), phenolics (p-hydroxy benzaldehyde, lignans), and sterols [9]. The root contains polysaccharides (glucans, glucogalacturonans, arabinogalactan acid) in large quantities, 6-methoxy-7-hydroxycoumarin (scopoletin), ceramides, monoterpenoids, and their glycosides, fatty triterpene, and phenylpropane (homovanillyl alcohol, and its 4-*O*-glycoside) [115,241,242] (Table 2).

#### 3.10.1. Preclinical Studies

Nettle root is recommended for relief of BPH and other prostate problems. It is also used for its cardiovascular effects and as a natural remedy to treat or prevent baldness [116,243,244,245,246]. It is proved that the polysaccharides and lectins prevent the prostate cellular metabolism and its growth [98]. Nettle extract inhibits TNF activity, while the lectins, malic acid, and caffeic acid show prostatic anti-proliferative and anti-inflammatory activities [30], due to the inhibition of COX and lipoxygenase [4]. However, it is important to emphasize that the polysaccharides stimulate the activity of T-lymphocytes and the complement activation [247]. Dreikorn [117] proved that the root extract can inhibit the connecting of a binding globulin (sex hormone binding globulin—SHBG) and its receptor in the membrane of the human prostatic cells. SHBG is a plasma protein that binds to sex hormones (estrogens and androgens), and regulates their plasma free fraction. The lignans contained in stinging nettle seem to have a high affinity for receptor of SHBG [30], and the extract inhibits prostate cell proliferation [116] (Table 2). The leaf extract has inhibitory effect against the activity of adenosine deaminase, which is the most important enzyme of the nucleotide metabolism. The inhibition is dose-dependent and it might be one of the mechanisms that leads to improvement of a patient’s symptoms [118]. Animal studies indicate that *U. dioica* markedly inhibits platelet aggregation and improves the lipid profile, due to the flavonoid content [248,249]. It was also found that the methanol extract of stinging nettle significantly inhibited the prostate growth induced experimentally [119,250]. The effects of stinging nettle on testosterone-induced BPH were investigated in rats. Simultaneous administration of petroleum ether and ethanolic extracts (10, 20 and 50 mg/kg^−1^ per os) and isolated β-sitosterol (10 and 20 mg/kg^−1^ per os) was undertaken. Measurement of prostate/body weight ratio, weekly urine output and serum testosterone levels, PSA levels (on day 28), and histological examinations carried out on prostates from each group allowed the conclusion that *U. dioica* can be used as an effective drug for the management of BPH [120].

#### 3.10.2. Clinical Studies

Clinical trials suggest a benefit of nettle extract for men with milder forms of BPH [251,252]. First, the effectiveness of a root extract (600 mg, 2 times/days/20 weeks) was demonstrated in the multicenter study of 4051 patients in various stages of BPH [253]. Friesen [254] reported the results in a multicenter long-term study for a total of 4480 patients who received nettle extract for 224 days on average, at doses of 600 mg twice/day for 3 months and then 600 mg daily during the remaining time. The extract improved urinary symptoms associated with BPH in 78% of patients after 3 months and in 91% of patients after 6 months. The diurnal and nocturnal urinary frequency was significantly improved, as well as the mean urine output [255]. Another randomized and double blind clinical study compared the effect of the aqueous root extract to placebo group. The dose was 120 mg; the patients used the root extract 3 times per day, for 6 months. Thanks to the treatment, the symptoms were relieved compared to the placebo group. The prevention effect of the treatment was identified after 18 months as well [256]. In an open-label extension of a randomized, double-blind clinical trial, the long-term efficacy and tolerability of a fixed combination of 160 mg Sabal fruit extract and 120 mg Urtica root extract per capsule (PRO 160/120) were investigated in elderly men with moderate or severe LUTS caused by BPH. Two hundred and fifty-seven patients were randomly treated with 2 × 1 capsule/day PRO 160/120 or placebo for 24 weeks, followed by a 24-week control period and a 48-week follow-up period in which all patients received PRO 160/120. It was concluded that the treatment with Sabal fruit and Urtica root provides a clinically relevant benefit over a period of 96 weeks [257]. Based on the previous clinical studies, it can be established that an increase in mean and maximum urinary flow rates and a reduction in PV and residual urine level were observed after treatment with nettle extract. Nettle root should be used for 6–12 months, as its use is possible for a long time without any serious adverse effects [255,258].

Adverse reactions could include mild gastrointestinal disturbances [57]. *U. dioica* contains tannins, which could interact with a concomitant intake of iron, causing a reduction of the effects in patients who need iron supplements. It is therefore, recommended to separate the times of administration of these components for at least 2 h. Edema and urticaria related to allergic reactions induced by the plant have been rarely reported [30].

## 4. Pharmaceutical Care

Men over the age of 45 are eligible for pharmaceutical management of BPH. There are three main target groups:

1. Men over 45 years of age who consult a pharmacist about lower urinary tract complaints;
2. Men over 45 years of age who purchase medication for the treatment of BPH;
3. Men over 45 years of age who have inadequate patient co-morbidity for BPH, in terms of medication management [11].

In all cases, there is a multi-step process involving pharmacist care for BPH.

The main points include the following:

1. Interviewing (in regard to the symptoms);
2. Questions related to the diagnosis;
3. Filling out the IPSS questionnaire;
4. Lifestyle advice;
5. Advice on OTC medicines;
6. Adherence (patient cooperation) questions;
7. Information on prescription medicines;
8. Referral to a doctor.

Patients in the first group have lower urinary tract complaints, and within this group, they can be further divided into subgroups.

1. Men with BPH-like symptoms. Patients should be interviewed about their symptoms, in this case, it is important to know if the urine stream is thinner, if it is intermittent or how often the patients have to get up to urinate at night. In addition, patients should be asked if they have ever seen a urologist or general practitioner about these symptoms. It is advisable at this time for patients to complete the IPSS questionnaire. This specific questionnaire based on the frequency and severity of symptoms can be used to assess the probability of mild, moderate, and severe BPH. Three categories of symptoms were described using the IPSS: mild 0–7, moderate 8–19, and severe 20–35. It is important to stress that an accurate diagnosis can only be made by a specialist after other tests have been carried out [10,11];
2. For patients diagnosed by a doctor with BPH, the pharmacist should inform the patient about safe medication use in the context of prescribed therapy. It is important to identify problems with safe medication use if they arise, and emphasis should be placed on increasing patient adherence;
3. There may be cases where patients present with other, non-BPH-like symptoms. Such cases are usually associated with alarm symptoms and should be referred to a doctor immediately. Alarm symptoms include painful urination, fever, bloody or cloudy urine in the last 3 months and urinary incontinence. These symptoms may indicate a urinary tract infection or chronic obstruction of the bladder. Thus, they definitely require medical attention.

Group 2 includes patients who buy BPH medication. When buying over-the-counter medicines, the pharmacist is obliged to inform patients about, for example, the indications, expected effects, and risks of herbal preparations. For prescription medicines, patients must also be informed and safety concerns identified, and information on lifestyle and over-the-counter products must be provided. With regard to lifestyle advice, it is recommended to reduce fluid intake to reduce the urge to urinate. Especially when the urge to urinate is most disturbing, e.g., at night or occasionally in public. Avoiding or reducing caffeine and alcohol consumption is also advisable. Regular exercise of the bladder is also suggested. This will increase the capacity of the bladder and may help to hold back urine when there is an urgent need to urinate.

Group 3 includes the non-adherent patients, who either do not take BPH medication or take them poorly.

In this case, the pharmacist should try to gain the patient’s trust and, thus, increase the patient’s adherence to the therapy.

Another important task for pharmacists is to detect drug interactions. Of the possible interactions, the effect on anticoagulant therapy is particularly important, as some drugs or herbal preparations may enhance the anticoagulant effects [11,12,13].

The issues outlined above illustrate the importance of pharmacists for the effectiveness of therapy.

## 5. Conclusions

The treatment of BPH is a complex process due to its multifactorial origins. Protocols of this disease include classic medical treatment, lifestyle and behavioral modifications, and phytotherapy. Moreover, regarding BPH research, researchers are focusing on new alternatives in the field of surgical treatment (e.g., water vapor thermal therapy), combinational therapies (e.g., combination of PDE5 inhibitors with 5α-reductase inhibitors, a β-3 adrenergic agonist with *α*1-adrenoceptor antagonists), and herbal supplements (particularly among the herbs of TCM, e.g., *Coptis chinensis)*. In mild or moderate cases, most patients ask for advice from a healthcare professional about non-prescription phytotherapeutics, or they choose a product on their own. In these cases, patients can be involved in the decision-making process, such as the choice of the pharmaceutical form. Herbal medicines are available in tablets, capsules, and oils, while synthetic medicines are only offered in tablet or capsule form. However, it is always important to advise the patient about the “category” of the product. This is crucial because traditional herbal medicines are regulated by the authorities, whereas dietary supplements are not. Another advantage of these natural products is that they can be easily combined with each other (e.g., saw palmetto fruit with pumpkin seed oil), to act on several targets at the same time. More serious conditions require medical prescriptions by a doctor. Because of the rapid onsets of action, first-line drug treatments include α1-blockers, 5α-reductase inhibitors, and their combinations, while herbal supplements require more time to achieve their effectiveness. It should be noted that well-tolerated phytotherapeutics showed fewer side effects and interactions by most patients in contrast to the above-mentioned medications. On the other hand, we should note that more information is needed about herb–drug and herb–herb interactions, to improve the safe use of these herbal supplements.

## Figures and Tables

**Table 1:** Most frequently used active compounds licensed in Europe in the treatment of LUTS [10,11,15].

Active Compound	Pharmacological Effect	Dose (mg)	Administration	Speed of Onset	Interaction May Occur
Alfuzosin	*α*1-adrenoceptor antagonist	7.5–10	Immediately following a meal, at the same time each day, depending on the formulation	days	In combination with vasodilators (e.g., PDE5 inhibitors, nitrates), and other antihypertensives. Before cataract surgery, consultation is necessary. In the case of tamsulosin, warfarin and diclofenac co-administration is not recommended.
Doxazosin	*α*1-adrenoceptor antagonist	2–8 (TR)	IR: daily once at bedtime ER: daily once with the first meal	days	
Silodosin	*α*1-adrenoceptor antagonist	4–8	With a meal, at the same time each day	days	
Tamsulosin	*α*1-adrenoceptor antagonist	0.4–0.8	30 min after the first meal	days	
Terazosin	*α*1-adrenoceptor antagonist	5–20 (TR)	Daily once at bedtime	days	
Dutasteride	5α-reductase (type 1 and 2) inhibitor	0.5	Without chewing, at the same time each day	6–12 months	In combination with strong CYP3A4 and CYP2D6 inhibitors.
Finasteride	5α-reductase (type 2) inhibitor	5	Without chewing, at the same time each day	6–12 months	No drug interactions have been identified.
Alfuzosin+ Finasteride	combination therapy	10/5	Swallow 2 different tablets without chewing, after dinner	days	ama
Tamsulosin+ Dutasteride	combination therapy	0.4/0.5	Swallow 1 tablet without chewing, 30 min after a meal, at the same time each day	days	ama

TR: titration recommended, IR: immediate release, ER: extended-release, ama: as mentioned above.

**Table 2:** Active compounds and biological activities of medicinal plants commonly used in the treatment of BPH, based on preclinical studies. Reference numbers provided in the brackets [ ].

Plant Species, Drug Part	Active Compounds	Biological Activities/Supposed Mechanism of Action
***Cucurbita pepo *** **seed**	Polysaccharides, sterols, para-aminobenzoic acid, proteins and peptides, carotenoids, γ-aminobutyric acid [34,35]; seed: fatty acids, phytosterols [36,37]	Inhibits 5α-reductase [50] Decrease of DHT level [56] Inhibits testosterone-induced hypertrophy [50] Antitumor [44]
***Epilobium*** ***parviflorum* and *E. angustifolium *** **aerial parts**	Polyphenols, steroids, triterpenoids, fatty acids [70]	Anti-inflammatory, antioxidative, anti-proliferative, antimicrobial, analgesic, anti-androgenic activities [70,71,72,73,74,75,76,77] Increase of CYP2D2 [78] Decrease of CYP3A1 expression [78] Decrease of CYP2E1 and CYP1A1 expression [79] Decrease of CYP2B1, CYP2C6, CYP2D2, CYP3A1 protein levels [80]
***Hypoxis hemerocallidea *** **corm**	Phytosterols: hypoxoside, rooperol, β-sitosterol, stigmasterol, stigmastanol; hypoxhemerolosides A–F, curcapicycloside, obtuside A, interjectin, crassifoside F, acuminoside, geraniol glycoside, vanillic acid, β-arbutin, orcinol glycoside [81,82]	Anti-inflammatory activity [4] Increase of TGF-β1 expression and protein kinase C-α activity in stromal cells [4]
***Solanum lycopersicum *** **fruit**	Tetraterpene carotenoids: lycopene, β-carotene, α-carotene; minerals, vitamins [83]	Antioxidant activity [84] Decreases the expression of nicotinamide adenine dinucleotide phosphate oxidase [85] Anticancer and anti-inflammatory activities [86]
***Pinus pinaster *** **bark**	Procyanidins, taxifolin, cinnamic acid, ferulic acid, caffeic acid, benzoic acid [87,88]	Anti-inflammatory activity [89,90] Nitric oxide synthase (eNOS) activity [89,90] Reduction in malondialdehyde, increased glutathione, catalase and peroxidase levels [91] Decrease of DHT level [92]
***Roystonea regia *** **fruit (oil)**	D-004 extract (oleic, lauric, palmitic and myristic acids) [4]	Inhibition of 5α-reductase [4] Antioxidant effects [4] Inhibition of phenylephrine-induced contractions in isolated rat prostate strips [93]
***Prunus africana *** **bark**	Phytosterols, fatty acids, triterpenes, proanthocyanidins, atraric acid, lauric acid, myristic acid, ferulic acid, atranorin, cholesterol, N-butylbenzene sulfonamide, hydroxybenzoic, linoleic, stearic, arachidonic, behenic, lignoceric acids [94,95,96,97,98,99]	Inhibits 5α-reductase [100,101], Inhibition of DHT and estrogen receptors [4], progesterone and androgen receptors [97]. Inhibits the basal growth of prostate stromal cells stimulated by EGF, IGF-I, bFGF, TPA, and PDBu [102]
***Secale cereale *** **pollen**	Carbohydrates, amino acids, proteins, phenolic compounds, sterols, triglycerides, plant pigments [103,104,105,106]	Inhibition of cyclooxygenase and the 5-lipoxygenase activity [107] Inhibition of prostaglandin and leukotriene synthesis [107] Anti-inflammatory activity—decreased TNF-α and IL-6 cytokines levels [108] Decreased androgen receptor and PSA expression [109]
***Serenoa repens *** **fruit**	Carbohydrates, sterols, flavonoids, triglycerides, fatty acids [110,111]	Inhibits 5α-reductase [111] >Inhibits formation of DHT and some testosterone metabolites [111] Inhibits the conversion of testosterone into DHT [111] Inhibition of α -receptor binding [111] Inhibits the receptor binding of androgens [111] Anti-proliferative effect [111] Inhibition of eicosanoid synthesis [111] Spasmolytic effects [111] Anti-inflammatory activity [111,112]
***Urtica dioica *** **root**	Sterols, flavonoids, tannins, acids, minerals, lectins, polysaccharides ceramides, monoterpenoids, fatty triterpene, and phenylpropane [4,9,113,114,115,116]	Anti-proliferative, anti-inflammatory—inhibition of COX and lipoxygenase [4] Inhibits TNF activity [4] Stimulates activity of T-lymphocytes and the complement activation [117] Inhibits the connecting of sex hormone binding globulin [118] Inhibits prostate growth [119,120]
